# Poetry Brings Out the Best in Us: Structured Poetry-Based Group Activity in Long-Term Care

**DOI:** 10.1093/geroni/igaa057.2423

**Published:** 2020-12-16

**Authors:** Daniel Kaplan, Gary Glazner

**Affiliations:** 1 Adelphi University, Cortlandt Manor, New York, United States; 2 The Alzheimer’s Poetry Project, New York, New York, United States

## Abstract

The Alzheimer’s Poetry Project has a proven track record at over 500 facilities in 35 states and six countries, serving over 50,000 people worldwide, and has the ability to bring high-quality creative arts programming to people living with dementia in long-term care. We describe how volunteers and recreation and care staff can be trained to deliver the intervention, offering direct benefits for participants and indirect benefits when modeled in the presence of providers and family members. Basic validation techniques are easily learned and incorporated into diverse dementia care strategies. As activity programs based in creative arts help to support self-expression among participants and serve as a vehicle for generating feelings of self-efficacy, we explain how such activities are well suited to fostering the person-centered goals of dementia care programming. Clinicians and other transdisciplinary care providers are encouraged to use and teach validation-focused arts interventions with persons living with dementia.

